# The physiological effects of acute and sub-lethal exposure to phenol on antioxidant enzyme activity in the freshwater sludge worm Tubifex tubifex

**DOI:** 10.1016/j.toxrep.2024.101717

**Published:** 2024-08-23

**Authors:** Debanjali Chakraborty, Ahamadul Hoque Mandal, Surajit Ghosh, Auroshree Sadhu, Debraj Das, Nimai Chandra Saha, Shubhajit Saha

**Affiliations:** a Ecotoxicology Research Laboratory, Department of Zoology, The University of Burdwan, Burdwan, West Bengal, India; b Centre for Computational and Data Sciences, Indian Institute of Technology, Kharagpur, West Bengal 721302, India; c Post Graduate Department of Zoology, Bidhannagar College, Sector 1, Bidhannagar, Kolkata, West Bengal 700064, India

**Keywords:** Phenol, *Tubifex tubifex*, Oxidative stress, Acute toxicity, Benthic annelid, Integrated biomarker response, Survival rate

## Abstract

The current study investigates the severe effects of commonly employed chemicals, such as phenol, on the freshwater bottom-dwelling annelids of *Tubifex tubifex*. In an acute toxicity test, phenol's 96-hour LC_50_ value against *Tubifex tubifex* was identified to be 221.552 mg/L. Using the GUTS simulation, which places the GUTS-SD model on top of the GUTS-IT model, it was possible to confirm that the test organism would survive an acute exposure to phenol overall. After 14 days of treatment with 10 % and 20 % of the phenol's 96-hour LC_50_ values, long-term bioassays revealed changes in protein levels and in oxidative stress enzyme levels. Total protein concentration dropped during the bioassay, but levels of antioxidant enzymes (CAT, GST, SOD, and MDA) increased. The Pearson correlation matrix and the Integrated Biomarker Response (IBR) index were used for examining the relationship between biomarkers, toxicants, and phenol-induced stress. The results show that exposure to phenol is detrimental to the survival and general health of *Tubifex tubifex.*

## Highlights

• The toxicity of the phenol was investigated in *Tubifex tubifex* in time dependent manner.
• Effects of phenol on oxidative stress enzymes is recorded in Oligochaete sludge worm.

## Graphical Abstract

**Figure:**
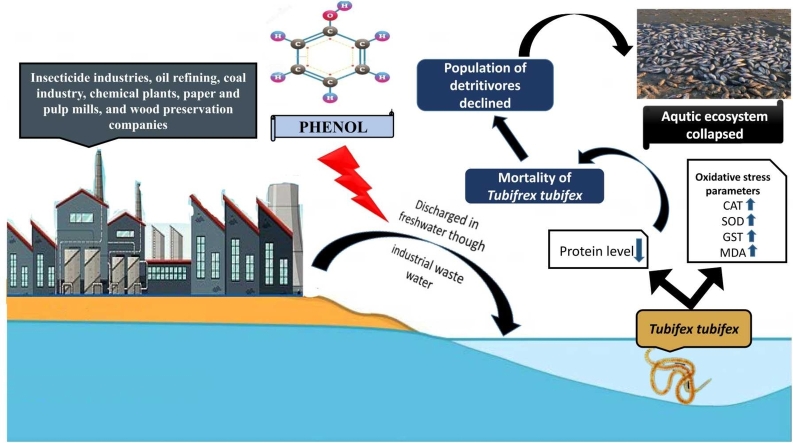

## 1 Introduction

Phenol is an aromatic organic molecule with the molecular formula C_6_H_5_OH. It is a volatile, colorless to white, crystalline solid with a characteristic odor [21]. Insecticides, dyes, coatings, oil refining, coal industry, chemical plants, paper and pulp mills, and wood preservation companies are among the many industries that extensively use it [14], [21], [30], [31], [4], [43], [48], [58]. Due to this widespread use, Phenol is common in effluent from numerous sectors [75]. Presently, phenol is in the International Maritime Organization's (IMO) list of the top 20 hazardous and noxious compounds [21]. According to the National Pollution Release Inventory, in 1996, 58 and 322 tonnes of phenol and its derivatives were discharged into the air and water respectively [13]. The European, Russian, and worldwide World Health Organization (WHO) set the maximum limit for phenol in water as 1 µg/litre [73]. However, previous data indicates that the highest concentration of phenol in surface waters was 2110 µg/L, while the highest phenol concentration in the final influent of the petroleum refinery sector is 3016 mg/L [14], [3]. As phenol is highly soluble in water (phenol, 8.28 g/100 ml), it can survive at high quantities in aquatic environments, and due to its lipophilicity, phenol is extensively circulated with metabolic products and is rapidly bioaccumulated [21].

When this toxicant enters the bloodstream, it can change various biomarkers or endpoints such as metabolic and biochemical endpoints [33], [41], [64], [69], histological endpoints [5], hematological endpoints, morphological and behavioural responses [1], [33], [54], [60], [65], neurological endpoints [5], [68], [70], Geno toxicological endpoints and immunological endpoints, which eventually lead to death. The structural modification of lipids, proteins, and nucleic acids inside cellular compartments is one of the harmful impacts of ROS, which may account for the change in a variety of biomarkers [16], [6]. Reactive oxygen species (ROS) are produced more readily by phenolic chemicals, which has been shown in previous studies to induce oxidative stress. García-Sánchez et al., [25]. The antioxidant defence system includes a variety of enzymes, such as Catalase (CAT), Glutathione-S-transferase (GST), Superoxide dismutase (SOD), Glutathione Peroxidase (GPx), and others. These antioxidant enzymes are crucial for maintaining cell homeostasis and acts as crucial biomarkers to detect oxidative stress [2], [32], [68], [7]. Consequently, the assessment of the antioxidant enzyme activities in the tissues reflect a tissue's vulnerability to an elevation in reactive oxygen species (ROS) generation and the related risks of the denaturation of protein, lipid peroxidation, apoptosis and DNA damage. Metrics such as integrated biomarker response (IBR) has been developed to lay out a precise assessment of the toxicant’s impact since biomarker interpretations are beneficial when used in conjunction with one another [42], [57], [62].Model-based approaches, such as the General Unified Threshold model for Survival (GUTS), offer an accurate method to calculate consequences of duration-dependent exposures to toxicants on the survival of aquatic organisms and resolve disputes in computing environmental risk evaluation in a more efficient way than experimental approaches do for ecologically relevant risk assessments [42].

There have been various studies on the toxicity of phenol and other phenolic compounds to fish and other aquatic species [21], [33], [39], [71], but no comprehensive and integrated biomarkers study on phenol toxicity against *Tubifex tubifex*, the fish-feed oligochaete worm is currently available. *Tubifex tubifex* acts as a good bioindicator species as they can tolerate several ecological factors and as they can easily grow in laboratory conditions they are chosen as test organisms in our study.

In the current investigation, we looked into the prospect that, under sub-chronic exposure conditions, phenol can affect the protein levels and oxidative stress markers in *Tubifex tubifex*. Hence, this research examined protein levels and oxidative stress biomarkers level in *T. tubifex* under sub-chronic exposures to phenol. To find out if the model better captured our findings on acute toxicity, we simulated the survivability across exposure concentrations by using the GUTS-SD and IT modeling techniques. The IBR and BRI were used to compile all the endpoints into a single index and evaluate an overall health status of *Tubifex tubifex* which are exposed to phenol.

## 2 Materials and methods

### 2.1 Test organism and its maintenance

A stock aquarium that contains non-chlorinated water (temperature 28.5 ± 0.5◦C, pH 7.8 ± 0.5, free CO2 15.2 ± 0.7 mg/l, dissolved oxygen 6± 0.2 mg/l, total alkalinity 184 ± 7.3 mg/l as CaCO3, hardness 122 ± 4.9 mg/l as CaCO3) and with constant ventilation (provided by a Bluepet BL-108 aerator) was used to acclimate the adult *Tubifex tubifex* for an entire day. The test system was then populated with test organisms that had a mean length of 12 mm ± 0.5 mm. The physiochemical characteristics of water remained constant during the experimental bioassay. This CaCO3 sample has the following parameters: temperature of 30 ± 0.6 C, pH of 7.6 ± 0.4, free CO2 of 15.2 ± 0.7 mg/l, dissolved oxygen of 6 ± 0.2 mg/l, total alkalinity of 184 ± 7.3 mg/l, and hardness of 122 ± 4.9 mg/l.

### 2.2 Test chemicals

Phenol in technical grade was supplied by Sigma-Aldrich, Chemicals Pvt. Ltd., and the remaining reagents by Sisco Research Laboratories (SRL), India. Since phenol is innately water dissolvable, 1 % w/v (1 gm/ 100 ml) stock solution was poised by dissolving phenol in distilled water.

### 2.3 Acute toxicity bioassay

During acute toxicity bioassay 250 ml glass beakers were used in three replicate and each glass beaker contained 200 ml of tap water along with 10 *Tubifex tubifex*. The static renewal method was followed during the bioassay. Test organisms were then exposed to different concentrations of phenol in addition to control group that contained normal tap water devoid of any toxicant. Final concentrations of toxicant were determined following 96 hours of preliminary range-finding investigations. The final phenol concentrations to which the test organisms were treated were 100, 200, 300, 400, 500, 600, 700, and 800 mg/L, with an untreated control group (0.00 mg/L). Mortality rates were recorded at 24, 48, 72, and 96 hours into the bioassay. Finney's probit analysis was used for the calculation of the toxicant's LC_50_ values, with log concentration acting as a dependent variable and probit acting as an independent variable. Finney [23].

### 2.4 Subchronic toxicity bioassay

As a result, the test organisms were exposed to two phenol concentrations (the T1 and T2 groups), such as 10 % and 20 % of its 96-hour LC_50_ value, respectively. Saha, Saha [61]. Phenol was added to test organisms T1 and T2 at concentrations of 22.15 and 44.30 mg/L, respectively. During the bioassay, a control group of Tubifex tubifex was maintained in parallel. In this bioassay, three groups (control, T_1,_ and T_2_) were kept in triplicate and the experiment was running for 14 days. On the first day of the experiment, the first treatments were administered. Every two days, the test medium was then refilled with matching toxicants at a concentration of 10 % of the initial amount. Saha, Saha [61]. Blue pet BL-108 aerator was used to offer continuous aeration during the exposure durations.

### 2.5 Obtaining and getting ready a tissue sample

At periodic intervals (1, 7, and 14 days), 1 g of the test organism was collected from each of the replicates and homogenized in a 0.1 M phosphate buffer solution (pH 7.6) [34]. The homogenate was then centrifuged at 10,000 rpm (Hermle Labortechnik, model no. Z36HK) for 10 minutes. The supernatant was then taken away and kept at −20 °C for further analysis later.

### 2.6 Protein content analysis

Utilizing bovine serum albumin as standard solution, the protein content was assessed by following the Bradford [12] protocol.

### 2.7 Oxidative stress enzymes analysis

The catalase enzyme activity (CAT) was assessed using a standard protocol [9]. The Beauchamp and Fridovich [8] method was employed to identify the superoxide dismutase (SOD) enzyme. Converting glutathione S-transferase (GSH) to 1-chloro-2,4-dinitrobenzene allowed researchers for measuring GST activity [27]. Activity of Glutathione peroxidase (GPx) was found, as per Lawrence and Burk (1976). Formation of thiobarbituric acid reactive substances (TBARS) made it possible to monitor the level of malondialdehyde (MDA). Ohkawa et al., [45]. Whereas the MDA level was represented as nmol TBARS/mg protein, the units of SOD, CAT, GST, GSH, and GPx were expressed as U/mg protein. All the parameters were measured using a UV–visible spectrophotometer (Cecil Aquarius CE 7400), at room temperature.

### 2.8 Determination of IBR

The "Integrated Biomarker Response" (IBR), a unified "stress assessment," was developed by the researchers by integrating all of the biomarker responses in a comprehensive perspective [10], [15], [19], [42], [52], [55]. Utilizing a modified equation provided by Samanta et al. [63] and reported by Beliaeff and Burgeot [10] the integrated biomarker response (IBR) was computed.

### 2.9 Statistical analysis

The LC_50_ values were calculated in Microsoft Excel 2013 using Finney's probit analysis. For data processing, GraphPad Prism v9 and Past 4.2 have been used. The mean ± SD is used to present all values. The Kaplan-Meier estimates provided the survival curves' base. The test organism’s survival rate pattern in response to phenol at the acute toxicity level was verified using the GUTS modelling carried out with the OpenGUTS® standalone program (Jager et al., 2011). After comparing the differences between the control and treatment groups using a two-way ANOVA, Tukey's post hoc analysis was conducted. The relationship between several biomarkers was visually represented using Pearson's correlation matrix. The study relied on the following values of statistical significance: p < 0.05, p < 0.01and p < 0.001.

## 3 Results and discussions

### 3.1 Acute toxicity of phenol against Tubifex tubifex

**Fig. 1:**
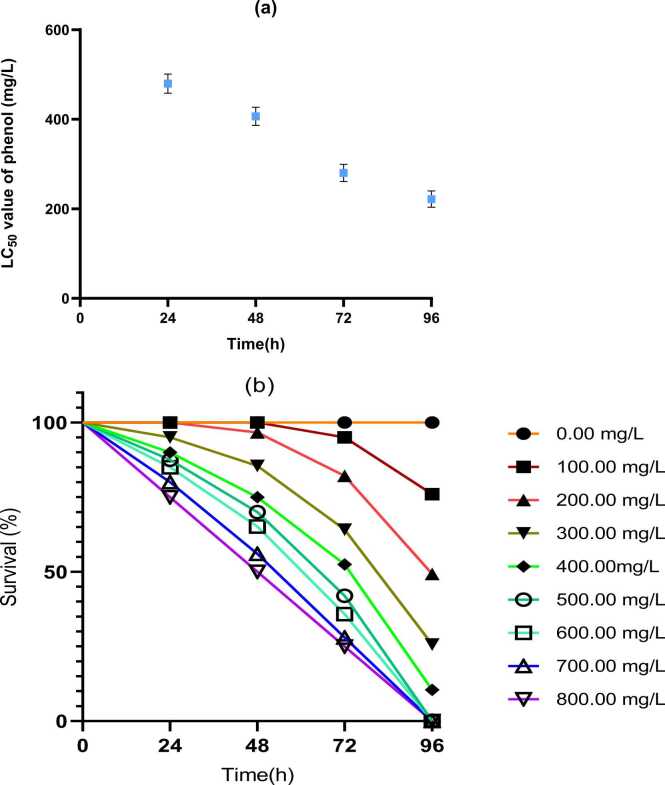
a-b. (a) 24, 48, 72, and 96 h LC_50_ values associated with 95 % confidence intervals of phenol and (b) Kaplan–Meier survival curves of *Tubifex tubifex* exposed to phenol (Log-rank Mantel-Cox test; Chi-square- 126.20; df 1; p-value: < 0.0001; **** = sig. difference between survival curves).

Table 1 and Fig. 1a both display the LC_50_ values of phenol to *Tubifex tubifex* at 24, 48, 72, and 96 hours along with 95 % confidence limits (upper and lower), correlation coefficients, and chi-squared values. In a dose- and time-dependent manner, the survival curve shows that phenol significantly reduces the overall survival rates of *Tubifex tubifex* (Mantel log-rank test; P < 0.05). (Fig. 1b). In the present study, *Tubifex tubifex* was found to be 100 % viable under control settings for every exposure time (24, 48, 72, and 96 hours) as well as for two exposure times (24 and 48 hours) at a concentration of 100 mg/L phenol. Nevertheless, the survival rate of *Tubifex tubifex* decreased significantly as the concentrations and exposure times (24, 48, 72, and 96 hours) of phenol increased. In this study, the 24, 48, 72, and 96 hours LC50 values of phenol were 479.56, 406.58, 280.06, and 221.55 mg/L, respectively. The type of test organisms, their size, age, and health, and the overall physiochemical characteristics of the water regulate the LC_50_ values for diverse aquatic organisms when exposed to toxicants (Sadat Sadeghi, 2018). When the computed phenol LC_50_ values for *Tubifex tubifex* are contrasted with the previous research conducted on different aquatic species [11], [13], [21], [3], [39], [5], [50], it is definite that *Tubifex tubifex* becomes vulnerable upon exposure to phenol.

**Table 1:** 24, 48, 72 and 96 h LC50 values of phenol against Tubifex tubifex along with 95 % confidence limits (upper and lower, correlation coefficients, and chi squared values, Graphpad Prism v8).

Exposure periods (h)	LC_50_ ± SE (mg/L)	95 % confidence limit		Correlation coefficient (r)	Chi squared value (χ^2^)
		Upper	Lower		
24	479.565	400.713	573.934	0.962	0.882^b^
48	406.581	340.108	486.046	0.966	0.999^b^
72	280.067	221.223	354.564	0.966	0.998^b^
96	221.552	167.991	292.188	0.962	0.921^b^

### 3.2 Estimating the probability of survival (GUTS)

**Fig. 2:**
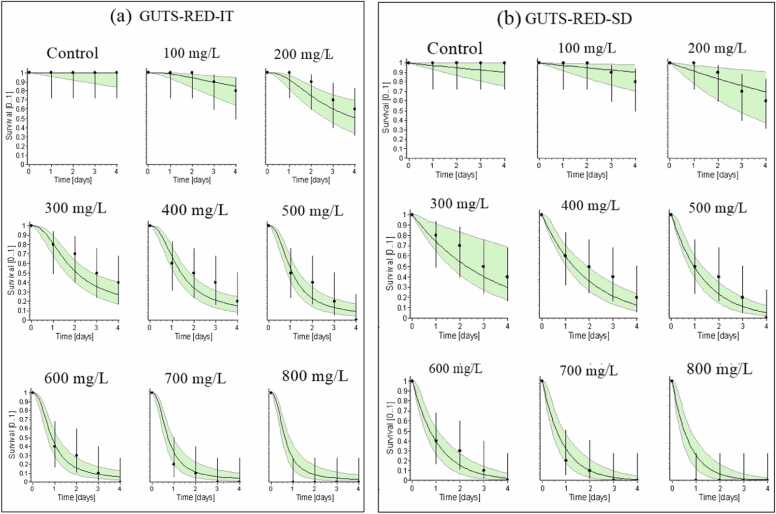
a-b. Relative fit of observed and fitted values of the (a) GUTS-SD (General Unified Threshold- Stochastic Death) and (b) GUTS-IT (General Unified Threshold- Individual Tolerance) models at different phenol exposure concentrations.

Fig. 2 illustrates the fitted performance of General Unified Threshold (Stochastic Death or Individual Tolerance) model. For *Tubifex tubifex*, the survival rate fits at 0.00 mg/L at all concentrations of exposure of phenol for the GUTS-IT model simulation, but is overestimated at 100, 200, 300, 400, 500, and 600 mg/L, and underestimated for 700 and 800 mg/L (Fig. 2a). The survival rates at 0.00, 300,400,500, and 600 mg/L of phenol in the GUTS-SD model show overestimation while underestimated at 700 and 800 mg/L (Fig. 2b). In contrast to GUTS-IT, GUTS-SD estimated larger values for all parameters. However, based on AIC values, GUTS-SD's fitting performance (AIC value = 189.36) was higher than GUTS-IT's (AIC value = 193.98) in the phenol instance. This suggests that the model simulation indicates that the GUTS-SD model is more accurate than the GUTS-IT model at predicting the survival rate in *Tubifex tubifex* for phenol exposure (Supplementary material). The survival model's findings demonstrate the importance of carefully choosing the model that infers SD or IT when examining the harmful effects of different toxicants. It is clear that this kind of mechanistic modeling holds enormous potential for improving environmental risk management in the future and could be very helpful in making informed decisions. Toxicodynamic recovery includes the physiological and cellular healing mechanisms that underpin an organism's stress response to toxic injury (Leist et al., 2017)[17], [67].

### 3.3 Alteration in protein level

**Fig. 3:**
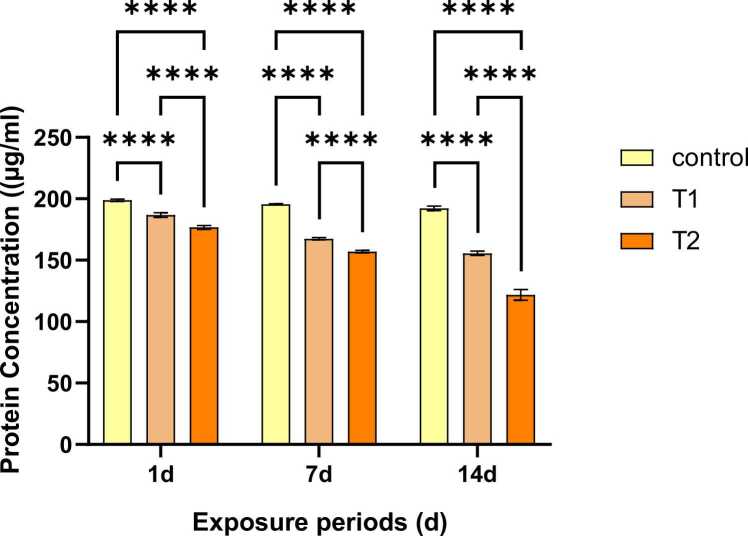
Alteration in protein level in *Tubifex tubifex* due to exposure at sublethal concentrations of phenol. The values are presented as mean ± SEM; ns indicates non-significant differences and the * indicates level of significance (*p < 0.05, **p < 0.01, ***p < 0.001 and ****p < 0.0001.

In this study, exposure of 10 % and 20 % of the 96-hour LC_50_ values of phenol led to a significant decline (P < 0.05) in protein concentration (µg/ml) at all exposure times (1d, 7d and 14d). (Fig. 3). The reduced plasma total soluble protein levels might be due to suppression of some specific cell-processing enzymes that regulate protein production which in turn interferes with protein synthesis [29].

### 3.4 Shift in the concentrations of antioxidant enzymes

**Fig. 4:**
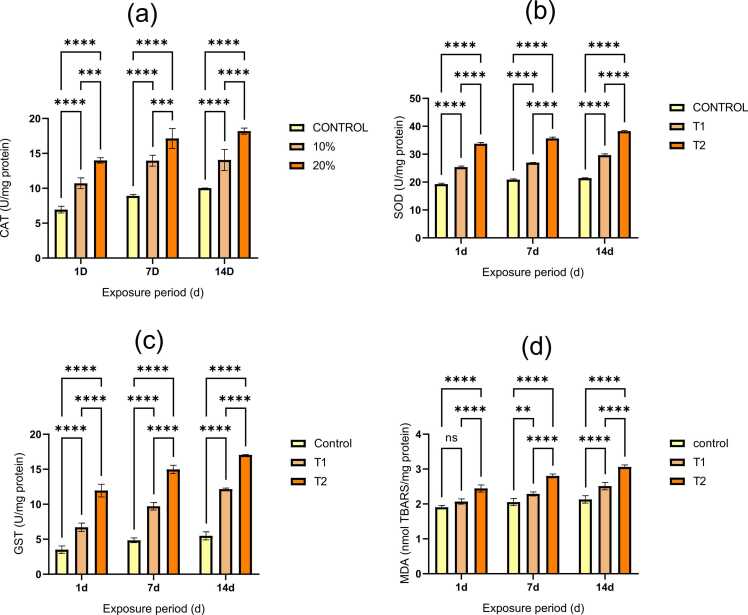
a-d. Alteration in oxidative stress parameters in *Tubifex tubifex* upon addition of phenol. Data are shown as mean ± SEM; ns denotes non-significant differences and the * denotes level of significance (**p < 0.01, ***p < 0.001 and ****p < 0.0001).

The alteration of oxidative stress enzymes in Tubifex tubifex after exposure to phenol is shown in Fig. 4. An antioxidative enzyme called superoxide dismutase (SOD) neutralizes free radicals like superoxide radicals (O) and shields cells from oxidative damage by converting superoxide radicals into hydrogen peroxide.

Hydrogen peroxide is broken down into water and oxygen by the antioxidant enzymes glutathione peroxidase (GPx) and catalase (CAT) [35], [46]. Since coordination between CAT and GPx stops radical intermediates from forming while reducing hydrogen peroxide and organic hydroperoxide, it is essential for the preservation of cell homeostasis. Ogueji et al., [44]. The catalytic integration of xenobiotic molecules with glutathione is facilitated by glutathione S-transferase (GST) enzyme, which is crucial for detoxification [38]. In this study, exposure of 10 % and 20 % 96-hour LC50 value significantly increases SOD and GST activity; however, exposure to 20 % of the 96-hour LC50 value during all exposure periods causes CAT activity to significantly decrease after initially increasing (1d, 7d, and 14d). Superoxide ion activation may be responsible for an increase in SOD activity because it encourages the production of SOD and shields cells from oxidative damage [40], [76]. In the current study, as organisms were exposed to the toxicant, CAT activity may have initially inclined because of a decrease in the harmful effects of higher ROS production that the toxicant was mediating [36] but the reduction in higher doses might be due to inhibitory effect of toxicants on CAT [24] or due to competition between GPx and CAT for the same substrate [26]. This study indicates that there is an increase in GST activity. One could interpret this event as a combined expression of the peroxidase-like isoform of GST and total GST [37]. Under stressful conditions, the body's defense mechanisms—such as antioxidant enzymes—cannot get rid of ROS when levels are high. ROS reacted with polyunsaturated fatty acids in cellular and organelle membranes in this stressed environment, hydrolyzing them to produce lipid peroxides [49]. Lipid peroxidation (LPO) byproducts like malondialdehyde (MDA) are regarded as indicators of higher cellular ROS levels and symptoms of cellular damage [22], [66]. In our study, MDA activity is significantly increased (P < 0.05) at all exposure durations (1d, 7d, and 14d) when exposed to 10 % and 20 % of the 96-hour LC50 value of phenol. The increase in MDA levels indicates a change in the permeability of cell membranes, allowing toxicants to enter the cell and finally cause apoptosis and DNA damage [72].

### 3.5 Correlation analysis amongst biomarkers

**Fig. 5:**
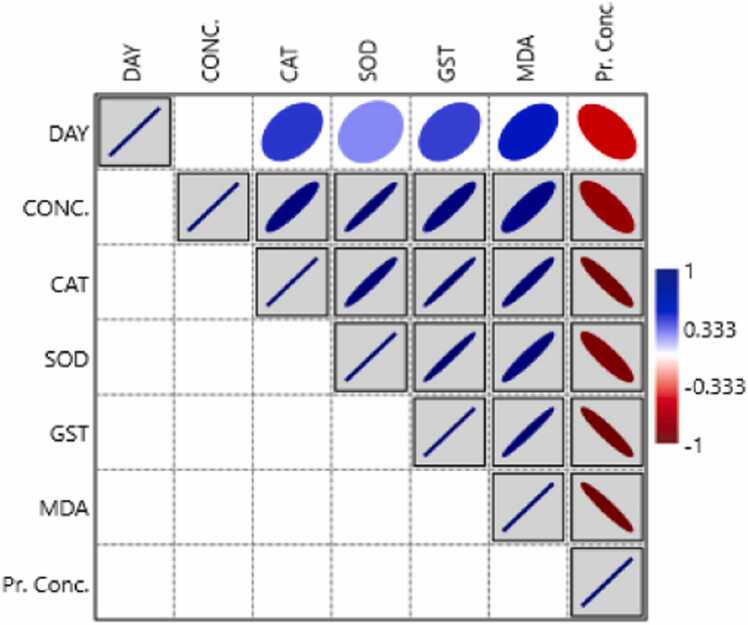
Pearson correlation matrix showing the significance levels between phenol exposure concentrations (mg/L), exposure durations (d), protein concentration (µg/L), and oxidative stress biomarkers in *Tubifex tubifex.*

The Pearson correlation test was utilized to predict an overall relationship between the biomarkers using phenol concentration (mg/l), time (d), protein concentration (µg/L), and oxidative stress biomarkers (CAT, SOD, GST, and MDA or LPO) (Fig. 5). The findings showed that the concentration of toxicants consistently correlated significantly negative (P <0.05) with the concentration of proteins, but significantly positively (p>0.05) with the levels of SOD, GST, CAT, and MDA. While CAT activity is significantly negatively correlated with protein concentration, it is substantially positively correlated with other oxidative stress biomarkers (GST, SOD, and MDA). Conversely, there is a significant inverse relationship (P < 0.05) between protein concentration and the phenol concentration (mg/L), oxidative stress biomarkers (CAT, GST, SOD, and MDA) activity, and exposure times (days).

### 3.6 Integrated biomarker response (IBR)

**Fig. 6:**
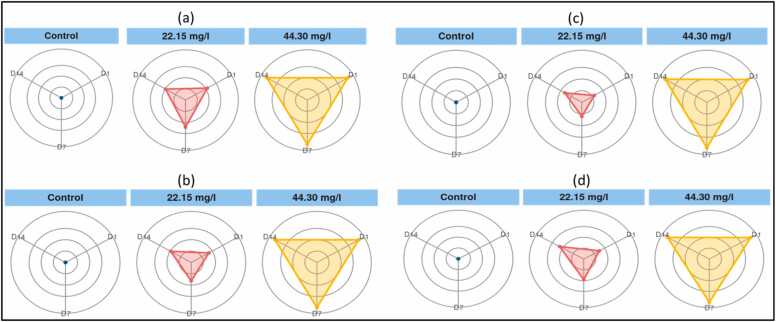
IBR star plots for evaluating oxidative stress biomarkers (a- CAT, b- SOD, c- MDA and d- GST).

Using more than one biomarker responses, the IBR method prospectively measures sensitivity of worms to toxicants [20], [35], [42]. The collective stress on *Tubifex tubifex* was calculated using the IBR index. These parameters (MDA, SOD, CAT and GST) have the largest reactions compared to other parameters; therefore, they are ideal for the IBR index [47]. The order of toxicity caused by phenol exposure, as illustrated by this index, is T2 > T1 > T0 (T2- 44.30 mg/L, T1- 22.15 mg/L, T0- control group). For all of the biomarkers under consideration, the converted data are depicted as star plots in Fig. 6 [18], [51], [53], [61]. The IBR method simplifies the process of describing a population's "total health state" by integrating the signals from multiple biomarkers. Several studies [56], [59], [61] confirm this.

### 3.7 In silico analysis

**Fig. 7:**
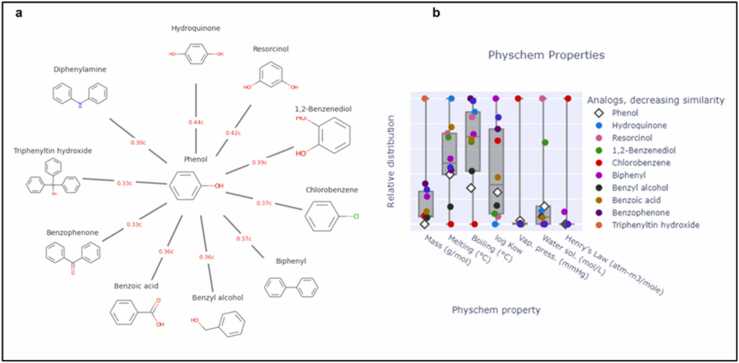
Baseline GenRA analysis of phenol using ToxRef data on Morgan fingerprints. (a) indicates ten nearest neighbouring chemicals that are analogous to phenol. They are arranged in a clockwise manner, while hydroquinone is showing the highest order of similarity and diphenylamine shows the least. Common hazardous substructures are marked with a different colour. (b) displaying neighbouring chemicals and their similarity level for different physiochemical properties with phenol.

Based on structural similarity, read-across can utilise source analogues to impute similar properties for a target substance. Generalized Read-Across (GenRA) uses a similarity-weighted average of source analogues described by their chemical and/or bioactivity descriptors to make read-across prediction of toxicity effects [74]. Here we performed GenRA analysis [28] of phenol to identify nearby or related analogous compounds. In Fig. 7a, the radial plot obtained using a baseline GenRA study depicts ten nearest chemicals that resemble phenol most. The nearest neighbours were filtered against ToxRef data and obtained using morgan fingerprinting. However, the analogous study based on Jaccard similarity identifies hydroquinone, a benzene derivative, as the most comparable member to phenol, while diphenylamine, a derivative of aniline, shows the least similarity. We can order the chemicals according to their similarity index with phenol in a decreasing manner as hydroquinone (0.44c), resorcinol (0.42c), 1,2-benzenediol (0.39c), chlorobenzene (0.37c), biphenyl (0.37c), benzyl alcohol (0.36c), benzoic acid (0.36c), benzophenone (0.33c), triphenyltin hydroxide (0.33c), diphenylamine (0.30c). Each of these chemicals possesses a common hazardous substructure marked with a different colour in Fig. 7b*.* All these neighbouring chemicals share many common physiochemical properties like mass (g/mol), melting point, boiling point, vapour pressure etc. Choosing a particular chemical for imputing a specific property can be carried out using the analysis done in Fig. 7b.

## 4 Conclusion

When *T. tubifex* was exposed to phenol, oxidative stress was induced as well as changed protein levels increased in concentration-dependent manner. Phenol has a high acute toxicity in wild, which is strongly corroborated by numerous antioxidant and oxidative stress responses which are observed in *T. tubifex* after 24 hours of exposure. In addition, greater metabolic disruption and ecological repercussions caused by phenol are reflected in the broader range of responses it induces. This article presents the first investigation on the lethality of phenol on *T. tubifex*, demonstrating time-dependent toxic effects. Although degeneration of integumentary and digestive tracts following aniline exposure raises concerns about tubifex's ability to persist in wild, it may also have severe effects on ecological systems in aquatic environments with fewer animals which consume detritus. Therefore, due to the fact that the distribution of detritus has an effect on the availability of dissolved nutrients as well as organic matter for biotic absorption, the slower breakdown of depositional resources may cause a reduction in the ecological performance of aquatic ecosystems.

## Ethical approval

Ethical clearance is not needed to study invertebrates like *Tubifex tubifex.*

## Funding sources

No government agency, business, or non-profit foundation provided financial support for this study

## Consent for publication

Not applicable.

## Consent to participate

Not applicable.

## CRediT authorship contribution statement

**Nimai Chandra Saha:** Writing – review & editing, Writing – original draft, Visualization, Supervision. **Shubhajit Saha:** Writing – review & editing, Writing – original draft, Investigation, Funding acquisition, Formal analysis, Data curation, Conceptualization. **Debanjali Chakraborty:** Writing – original draft, Methodology, Investigation, Formal analysis, Data curation. **Ahamadul Hoque Mandal:** Writing – original draft, Software, Resources, Methodology, Investigation, Formal analysis, Data curation. **Surajit Ghosh:** Writing – original draft, Methodology, Investigation, Funding acquisition, Formal analysis, Data curation. **Auroshree Sadhu:** Writing – original draft, Software, Methodology, Investigation, Formal analysis, Data curation. **Debraj Das:** Writing – original draft, Software.

## Declaration of Competing Interest

The authors declare that they have no known competing financial interests or personal relationships that could have appeared to influence the work reported in this paper.

## Data Availability

Data will be made available on request.

## References

[1] Aliko V., Vasjari L., Ibrahimi E., Impellitteri F., Karaj A., Gjonaj G., Piccione G., Arfuso F., Faggio C., Istifli E.S. (2024). From shadows to shores”-quantitative analysis of CuO nanoparticle-induced apoptosis and DNA damage in fish erythrocytes: a multimodal approach combining experimental, image-based quantification, docking and molecular dynamics. Sci. Total Environ..

[2] Aljahdali M.O., Alhassan A.B. (2020). Metallic pollution and the use of antioxidant enzymes as biomarkers in Bellamya unicolor (Olivier, 1804)(Gastropoda: Bellamyinae). Water.

[3] Ayeni O. (2014). A preliminary assessment of phenol contamination of Isebo River in south-western Nigeria. Greener J. Phys. Sci..

[4] Azadikhah D., Varcheh M., Yalsuyi A.M., Forouhar Vajargah M., Mansouri Chorehi M., Faggio C. (2023). Hematological and histopathological changes of juvenile grass carp (CtenopharyngoDon Idella) exposed to lethal and sublethal concentrations of roundup (Glyphosate 41% SL). Aquac. Res..

[5] Babich H., Davis D. (1981). Phenol: a review of environmental and health risks. Regul. Toxicol. Pharmacol..

[6] Banaee M., Faraji J., Amini M., Multisanti C.R., Faggio C. (2023). Rainbow trout (Oncorhynchus mykiss) physiological response to microplastics and enrofloxacin: novel pathways to investigate microplastic synergistic effects on pharmaceuticals. Aquat. Toxicol..

[7] Banaee M., Impellitteri F., Multisanti C.R., Sureda A., Arfuso F., Piccione G., Faggio C. (2023). Evaluating silymarin extract as a potent antioxidant supplement in diazinon-exposed rainbow trout: oxidative stress and biochemical parameter analysis. Toxics.

[8] Beauchamp C., Fridovich I. (1971). Superoxide dismutase: improved assays and an assay applicable to acrylamide gels. Anal. Biochem..

[9] Beers R.F., Sizer I.W. (1952). A spectrophotometric method for measuring the breakdown of hydrogen peroxide by catalase. J. Biol. Chem..

[10] Beliaeff B., Burgeot T. (2002). Integrated biomarker response: a useful tool for ecological risk assessment. Environ. Toxicol. Chem.: Int. J..

[11] Bettinetti R., Provini A. (2002). Toxicity of 4-nonylphenol to Tubifex tubifex and Chironomus riparius in 28-day whole-sediment tests. Ecotoxicol. Environ. Saf..

[12] Bradford M.M. (1976). A rapid and sensitive method for the quantitation of microgram quantities of protein utilizing the principle of protein-dye binding. Anal. Biochem..

[13] Breton R.L., Teed R.S., Moore D.R. (2003). An ecological risk assessment of phenol in the aquatic environment. Hum. Ecol. Risk Assess..

[14] Buikema A.L., McGinniss M.J., Cairns J. (1979). Phenolics in aquatic ecosystems: a selected review of recent literature. Mar. Environ. Res..

[15] Chakraborty D., Saha S., Podder S., Saha N.C., Faggio C. (2024). Generation of oxidative stress in freshwater oligochaete Tubifex tubifex due to exposure to aniline: time and concentration dependent toxicity assessment. Chem. Ecol.:1-16. doi.

[16] Chowdhury S., Saikia S. (2020). Oxidative stress in fish: a review. J. Sci. Res..

[17] Chukwuka A.V., Saha S., Mukherjee D., Banerjee P., Dhara K., Saha N.C. (2022). Deltamethrin-induced respiratory and behavioral effects and adverse outcome pathways (AOP) in short-term exposed mozambique tilapia, oreochromis mossambicus. Toxics.

[18] Dhara K., Das U.N., Prasenjit P., Saha N.C., Shubhajit S. (2023). Temperature-optimized, hormone-induced spawning of Asian striped dwarf catfish, Mystus vittatus in early-stage F1 generation. Iran. J. Ichthyol..

[19] Dhara K., Saha S., Chukwuka A.V., Pal P., Saha N.C., Faggio C. (2021). Fluoride sensitivity in freshwater snail, Bellamya bengalensis (Lamarck, 1882): an integrative biomarker response assessment of behavioral indices, oxygen consumption, haemocyte and tissue protein levels under environmentally relevant exposure concentrations. Environ. Toxicol. Pharmacol..

[20] Dhara K., Saha S., Pal P., Chukwuka A.V., Panigrahi A.K., Saha N.C., Faggio C. (2021). Biochemical, physiological (haematological, oxygen-consumption rate) and behavioural effects of mercury exposures on the freshwater snail, Bellamya bengalensis. Comp. Biochem. Physiol. Part C: Toxicol. Pharmacol..

[21] Duan W., Meng F., Cui H., Lin Y., Wang G., Wu J. (2018). Ecotoxicity of phenol and cresols to aquatic organisms: a review. Ecotoxicol. Environ. Saf..

[22] Faheem M., Lone K.P. (2018). Oxidative stress and histopathologic biomarkers of exposure to bisphenol-A in the freshwater fish, Ctenopharyngodon idella. Braz. J. Pharm. Sci..

[23] Finney D. (1971). Statistical logic in the monitoring of reactions to therapeutic drugs. Methods Inf. Med..

[24] Freitas R., Silvestro S., Coppola F., Costa S., Meucci V., Battaglia F., Intorre L., Soares A.M., Pretti C., Faggio C. (2020). Toxic impacts induced by Sodium lauryl sulfate in Mytilus galloprovincialis. Comp. Biochem. Physiol. Part A: Mol. Integr. Physiol..

[25] García-Sánchez M., Garrido I., Casimiro I. de Jesús, Casero P.J., Espinosa F., García-Romera I., Aranda E. (2012). Defence response of tomato seedlings to oxidative stress induced by phenolic compounds from dry olive mill residue. Chemosphere.

[26] Guyton K.Z., Xu Q., Holbrook N.J. (1996). Induction of the mammalian stress response gene GADD153 by oxidative stress: role of AP-1 element. Biochem. J..

[27] Habig W.H., Pabst M.J., Jakoby W.B. (1974). Glutathione S-transferases: the first enzymatic step in mercapturic acid formation. J. Biol. Chem..

[28] Helman G., Shah I., Williams A.J., Edwards J., Dunne J., Patlewicz G. (2019). Generalised read-across (GenRA): a workflow implemented into the EPA CompTox chemicals dashboard. Altex.

[29] Ibrahim N.M., Eweis E.A., El-Beltagi H.S., Abdel-Mobdy Y.E. (2012). Effect of lead acetate toxicity on experimental male albino rat. Asian Pac. J. Trop. Biomed..

[30] Impellitteri F., Riolo K., Multisanti C.R., Zicarelli G., Piccione G., Faggio C., Giannetto A. (2024). Evaluating quaternium-15 effects on Mytilus galloprovincialis: new insights on physiological and cellular responses. Sci. Total Environ..

[31] Impellitteri F., Yunko K., Calabrese G., Porretti M., Martyniuk V., Gnatyshyna L., Nava V., Potortì A.G., Piccione G., Di Bella G. (2024). Chlorpromazine's impact on Mytilus galloprovincialis: a multi-faceted investigation. Chemosphere.

[32] Impellitteri F., Yunko K., Martyniuk V., Khoma V., Piccione G., Stoliar O., Faggio C. (2023). Cellular and oxidative stress responses of Mytilus galloprovincialis to chlorpromazine: implications of an antipsychotic drug exposure study. Front. Physiol..

[33] Inyang I.R., Izah S.C., Suobo K. (2019). Effect of phenol on the kidney and liver biochemical and metabolites of Clarias gariepinus. Ecotoxicology.

[34] Kaletaş B.K., van der Wiel I.M., Stauber J., Dekker L.J., Güzel C., Kros J.M., Luider T.M., Heeren R.M. (2009). Sample preparation issues for tissue imaging by imaging MS. Proteomics.

[35] Kim J.-H., Choi H., Sung G., Seo S.-A., Kim K.I., Kang Y.J., Kang J.-C. (2019). Toxic effects on hematological parameters and oxidative stress in juvenile olive flounder, Paralichthys olivaceus exposed to waterborne zinc. Aquac. Rep..

[36] Kumari K., Khare A., Dange S. (2014). The applicability of oxidative stress biomarkers in assessing chromium induced toxicity in the fish Labeo rohita. BioMed. Res. Int..

[37] Liu B., Yu Z., Song X., Yang F. (2010). Effects of sodium dodecylbenzene sulfonate and sodium dodecyl sulfate on the Mytilus galloprovincialis biomarker system. Ecotoxicol. Environ. Saf..

[38] Livingstone D. (1998). The fate of organic xenobiotics in aquatic ecosystems: quantitative and qualitative differences in biotransformation by invertebrates and fish. Comp. Biochem. Physiol. Part A: Mol. Integr. Physiol..

[39] Lv Y.-Z., Yao L., Wang L., Liu W.-R., Zhao J.-L., He L.-Y., Ying G.-G. (2019). Bioaccumulation, metabolism, and risk assessment of phenolic endocrine disrupting chemicals in specific tissues of wild fish. Chemosphere.

[40] Majumdar N., Chandra Saha N., Banerjee P., Bhattacharya T., Saha S. (2023). Acute and sub-acute toxic effects of cadmium to freshwater tropical oligochaete Tubifex tubifex with special reference to oxidative stress and behavioural biomarkers. Chem. Ecol..

[41] Mandal A.H., Ghosh S., Adhurjya D., Chatterjee P., Samajdar I., Mukherjee D., Dhara K., Saha N.C., Piccione G., Multisanti C.R., Saha S., Faggio C. (2024). Exploring the impact of zinc oxide nanoparticles on fish and fish-food organisms: a review. Aquac. Rep..

[42] Mukherjee D., Saha S., Chukwuka A.V., Ghosh B., Dhara K., Saha N.C., Pal P., Faggio C. (2022). Antioxidant enzyme activity and pathophysiological responses in the freshwater walking catfish, Clarias batrachus Linn under sub-chronic and chronic exposures to the neonicotinoid, Thiamethoxam®. Sci. Total Environ..

[43] Multisanti C.R., Riolo K., Impellitteri F., Chebbi I., Faggio C., Giannetto A. (2023). Short-term in vitro exposure of Pinctada imbricata’s haemocytes to quaternium-15: exploring physiological and cellular responses. Environ. Toxicol. Pharmacol..

[44] Ogueji E., Nwani C., Mbah C., Iheanacho S., Nweke F. (2020). Oxidative stress, biochemical, lipid peroxidation, and antioxidant responses in Clarias gariepinus exposed to acute concentrations of ivermectin. Environ. Sci. Pollut. Res..

[45] Ohkawa H., Ohishi N., Yagi K. (1979). Assay for lipid peroxides in animal tissues by thiobarbituric acid reaction. Anal. Biochem..

[46] Pandey S., Ahmad I., Parvez S., Bin-Hafeez B., Haque R., Raisuddin S. (2001). Effect of endosulfan on antioxidants of freshwater fish Channa punctatus Bloch: 1. Protection against lipid peroxidation in liver by copper preexposure. Arch. Environ. Contam. Toxicol..

[47] Paul T., Kumar S., Shukla S., Pal P., Kumar K., Poojary N., Biswal A., Mishra A. (2020). A multi-biomarker approach using integrated biomarker response to assess the effect of pH on triclosan toxicity in Pangasianodon hypophthalmus (Sauvage, 1878). Environ. Pollut..

[48] Ramya S., Barathinivas A., Jayakumararaj R., Pothiraj C., Ali D., Piccione G., Multisanti C.R., Balaji P., Faggio C. (2023). Ecotoxicological insights: effects of pesticides on ionic metabolism regulation in freshwater catfish, Mystus keletius. Aquat. Toxicol..

[49] Regoli F., Giuliani M.E. (2014). Oxidative pathways of chemical toxicity and oxidative stress biomarkers in marine organisms. Mar. Environ. Res..

[50] Saha N., Bhunia F., Kaviraj A. (1999). Toxicity of phenol to fish and aquatic ecosystems. Bull. Environ. Contam. Toxicol..

[51] Saha S., Chukwuka Azubuike V., Mukherjee Dip, Dhara Kishore, Adeogun A.O., Saha N.C. (2022). Effects of short-term sub-lethal diazinon® exposure on behavioural patterns and respiratory function in Clarias batrachus: inferences for adaptive capacity in the wild. Chem. Ecol. doi.

[52] Saha S., Chandra Saha N., Chatterjee A., Banerjee P., Garai P., Sharma P., Patnaik L., Nayak S., Dhara K., Chukwuka A., Faggio C. (2023). Integrated multi-biomarker responses in Mozambique tilapia, Oreochromis mossambicus under acute and chronic Diazinon® exposures. Chem. Ecol.:1-21. doi.

[53] Saha S., Chukwuka A.V., Mukherjee D., Dhara K., Pal P., Saha N.C. (2022). Physiological (haematological, growth and endocrine) and biochemical biomarker responses in air-breathing catfish, Clarias batrachus under long-term Captan® pesticide exposures. Environ. Toxicol. Pharmacol. doi.

[54] Saha S., Chukwuka A.V., Mukherjee D., Dhara K., Saha N.C., Faggio C. (2022). Behavioral and physiological toxicity thresholds of a freshwater vertebrate (Heteropneustes fossilis) and invertebrate (Branchiura sowerbyi), exposed to zinc oxide nanoparticles (nZnO): A General Unified Threshold model of Survival (GUTS). Comp. Biochem. Physiol. Part C: Toxicol. Pharmacol..

[55] Saha S., Chukwuka A.V., Mukherjee D., Patnaik L., Nayak S., Dhara K., Saha N.C., Faggio C. (2021). Chronic Effects of Diazinon® exposures using integrated biomarker responses in freshwater walking catfish, Clarias batrachus. Appl. Sci..

[56] Saha S., Dhara K., Pal P., Saha N.C., Faggio C., Chukwuka A.V. (2022). Longer-term adverse effects of selenate exposures on hematological and serum biochemical variables in air-breathing fish channa punctata (Bloch, 1973) and non-air breathing fish ctenopharyngodon idella (Cuvier, 1844): an Integrated Biomarker Response Approach. Biol. Trace Elem. Res..

[57] Saha S., Mukherjee D., Dhara K., Saha N.C. (2020). Captan-induced toxicity and behavioural alterations on oligochaete worm, branchiura sowerbyi. J. Aquat. Biol. Fish..

[58] Saha S., Mukherjee D., Dhara K., Saha N.C. (2021). Acute toxicity bioassay of a pyrethroid pesticide bifenthrin to the asian stinging catfish, heteropneustes fossilis (Bloch). Curr. World Environ..

[59] Saha, S., D. Mukherjee, K. Dhara, and N.C. Saha. 2022e. Acute toxicity bioassay of a pyrethroid pesticide bifenthrin to the Asian stinging catfish, Heteropneustes fossilis (Bloch).

[60] Saha S., Mukherjee D., Saha N.C. (2018). Studies on acute toxicity and behavioral responses of Heteropneustes fossilis (Linn.) exposed to Diazinon. Res. Rev.: A J. Toxicol..

[61] Saha S., Saha N.C. (2021). Study on acute toxicity of bifenthrin to Clarias batrachus (linn.). Indian J. Ecol..

[62] Saha S., Saha S., Mistri A., Saha N.C. (2023). Antioxidant enzyme activity and pathophysiological consequences in the sludge worm Tubifex tubifex under acute and sub-lethal exposures to the fungicide Tilt®. Pestic. Biochem. Physiol..

[63] Samanta P., Im H., Na J., Jung J. (2018). Ecological risk assessment of a contaminated stream using multi-level integrated biomarker response in Carassius auratus. Environ. Pollut..

[64] Sehonova P., Plhalova L., Blahova J., Doubkova V., Marsalek P., Prokes M., Tichy F., Skladana M., Fiorino E., Mikula P. (2017). Effects of selected tricyclic antidepressants on early-life stages of common carp (Cyprinus carpio). Chemosphere.

[65] Sehonova P., Svobodova Z., Dolezelova P., Vosmerova P., Faggio C. (2018). Effects of waterborne antidepressants on non-target animals living in the aquatic environment: a review. Sci. Total Environ. 631.

[66] Sharbidre A.A., Metkari V., Ka Patode P. (2011). Effect of diazinon on acetylcholinesterase activity and lipid. Res. J. Environ. Toxicol..

[67] Sharma P., Garai P., Banerjee P., Saha S., Chukwuka A.V., Chatterjee S., Saha N.C., Faggio C. (2023). Behavioral toxicity, histopathological alterations and oxidative stress in Tubifex tubifex exposed to aromatic carboxylic acids-acetic acid and benzoic acid: A comparative time-dependent toxicity assessment. Sci. Total Environ..

[68] Shiry N., Alavinia S.J., Impellitteri F., Alavinia S.J., Faggio C. (2023). Beyond the surface: Consequences of methyl tert-butyl ether (MTBE) exposure on oxidative stress, haematology, genotoxicity, and histopathology in rainbow trout. Sci. Total Environ..

[69] Shiry N., Darvishi P., Gholamhossieni A., Pastorino P., Faggio C. (2023). Exploring the combined interplays: effects of cypermethrin and microplastic exposure on the survival and antioxidant physiology of Astacus leptodactylus. J. Contam. Hydrol..

[70] Shiry N., Derakhshesh N., Alavinia S.J., Pouladi M., Falco F., Faggio C. (2023). Anodonta cygnea, a freshwater swan mussel, exposed to diazinon: toxicity thresholds in behaviour and physiology. Vet. Res. Commun..

[71] Singh A.K., Kumar A., Chandra R. (2020). Detection of refractory organic pollutants from pulp paper mill effluent and their toxicity on Triticum aestivum; Brassica campestris and Tubifex-tubifex. J. Exp. Biol. Agric. Sci..

[72] Song P., Gao J., Li X., Zhang C., Zhu L., Wang J., Wang J. (2019). Phthalate induced oxidative stress and DNA damage in earthworms (Eisenia fetida). Environ. Int..

[73] Stöfen D. (1973). The maximum permissible concentrations in the USSR for harmful substances in drinking water.. Toxicology.

[74] Tate T., Wambaugh J., Patlewicz G., Shah I. (2021). Repeat-dose toxicity prediction with Generalized Read-Across (GenRA) using targeted transcriptomic data: a proof-of-concept case study. Comput. Toxicol..

[75] Yan J., Jianping W., Jing B., Daoquan W., Zongding H. (2006). Phenol biodegradation by the yeast Candida tropicalis in the presence of m-cresol. Biochem. Eng. J..

[76] Zhang Q., Zhu L., Wang J., Xie H., Wang J., Han Y., Yang J. (2013). Oxidative stress and lipid peroxidation in the earthworm Eisenia fetida induced by low doses of fomesafen. Environ. Sci. Pollut. Res..

